# Automated detection of cervical ossification of the posterior longitudinal ligament in plain lateral radiographs of the cervical spine using a convolutional neural network

**DOI:** 10.1038/s41598-021-92160-9

**Published:** 2021-06-16

**Authors:** Masataka Miura, Satoshi Maki, Kousei Miura, Hiroshi Takahashi, Masayuki Miyagi, Gen Inoue, Kazuma Murata, Takamitsu Konishi, Takeo Furuya, Masao Koda, Masashi Takaso, Kenji Endo, Seiji Ohtori, Masashi Yamazaki

**Affiliations:** 1 Department of Orthopaedic Surgery, Chiba University Graduate School of Medicine, 1-8-1 Inohana, Chuou-ku, Chiba, 260-8670 Japan; 2 Department of Orthopaedic Surgery, Faculty of Medicine, University of Tsukuba, Tsukuba, Japan; 3 Department of Orthopaedic Surgery, Kitasato University School of Medicine, Sagamihara, Japan; 4 Department of Orthopedic Surgery, Tokyo Medical University, Tokyo, Japan

**Keywords:** Machine learning, Spinal cord diseases, Spine regulation and structure

## Abstract

Cervical ossification of the posterior longitudinal ligament (OPLL) is a contributing factor to spinal cord injury or trauma-induced myelopathy in the elderly. To reduce the incidence of these traumas, it is essential to diagnose OPLL at an early stage and to educate patients how to prevent falls. We thus evaluated the ability of our convolutional neural network (CNN) to differentially diagnose cervical spondylosis and cervical OPLL. We enrolled 250 patients with cervical spondylosis, 250 patients with cervical OPLL, and 180 radiographically normal controls. We evaluated the ability of our CNN model to distinguish cervical spondylosis, cervical OPLL, and controls, and the diagnostic accuracy was compared to that of 5 board-certified spine surgeons. The accuracy, average recall, precision, and F1 score of the CNN for classification of lateral cervical spine radiographs were 0.86, 0.86, 0.87, and 0.87, respectively. The accuracy was higher for CNN compared to any expert spine surgeon, and was statistically equal to 4 of the 5 experts and significantly higher than that of 1 expert. We demonstrated that the performance of the CNN was equal or superior to that of spine surgeons.

## Introduction

Ossification of the posterior longitudinal ligament (OPLL) is characterized by ectopic bone formation within the posterior longitudinal ligament of the spine. The prevalence of OPLL in Japan has been reported to be 1.9–4.3% for people over the age of 30, 1.0–3.0% in Asian countries such as China and South Korea, and 0.1–1.7% in continental Europe and North America^1–3^. OPLL of the cervical spine is a contributing factor to spinal cord injury and trauma-induced myelopathy in the elderly, and there is a need to educate patients with OPLL to prevent falls^4–7^. Therefore, early detection of OPLL is crucial to avoid spinal cord injury or trauma-induced myelopathy due to OPLL. Although the widespread use of computed tomography (CT) revealed that the diagnostic accuracy of a simple cervical radiograph was inadequate^8^, radiographs are still a mainstay for screening cervical spine pathology.

Convolutional neural network (CNN) has been developed to mimic the central nervous system in human image recognition, and it automatically and adaptively learns features from data using multiple building blocks^9^. Notably, CNN is an artificial intelligence technique that is useful in the field of image recognition, including for medical imaging. To date, however, artificial intelligence has had only limited applications in spinal diseases.

The purpose of this study is to determine whether it is possible to make a differential diagnosis of cervical spondylosis and cervical OPLL using a CNN. We also compare the diagnostic accuracy of the CNN with that of expert spine surgeons to verify whether the CNN can serve as a screening tool for OPLL.

## Results

### Patient characteristics

The characteristics of the patients enrolled in this study are shown in Table 1. Patients with no abnormal radiographic findings consisted mostly of individuals with whiplash and neck pain. The group of patients with cervical spondylosis included 236 cases of cervical spondylotic myelopathy and 14 cases of cervical spondylotic radiculopathy. Among the test dataset of the OPLL patients, the distribution of the OPLL type was 6 patients with the continuous type, 18 with the segmental type, 22 with the mixed type, and 4 with the localized type.

**Table 1:** Baseline patient characteristics.

	Cervical spondylosis	OPLL	Normal
n (patients)	250	250	180
Age	65.6 ± 12.2	64.0 ± 11.0	24.0 ± 5.4
Sex (M/F)	174/76	183/67	95/85
JOA score	8.8 ± 3.6 (n = 240)	11.5 ± 4.0 (n = 233)	–

*JOA* Japanese Orthopaedic Association, *OPLL* ossification of posterior longitudinal ligament.

### Performance of the CNN and spine surgeons

**Figure 1:**
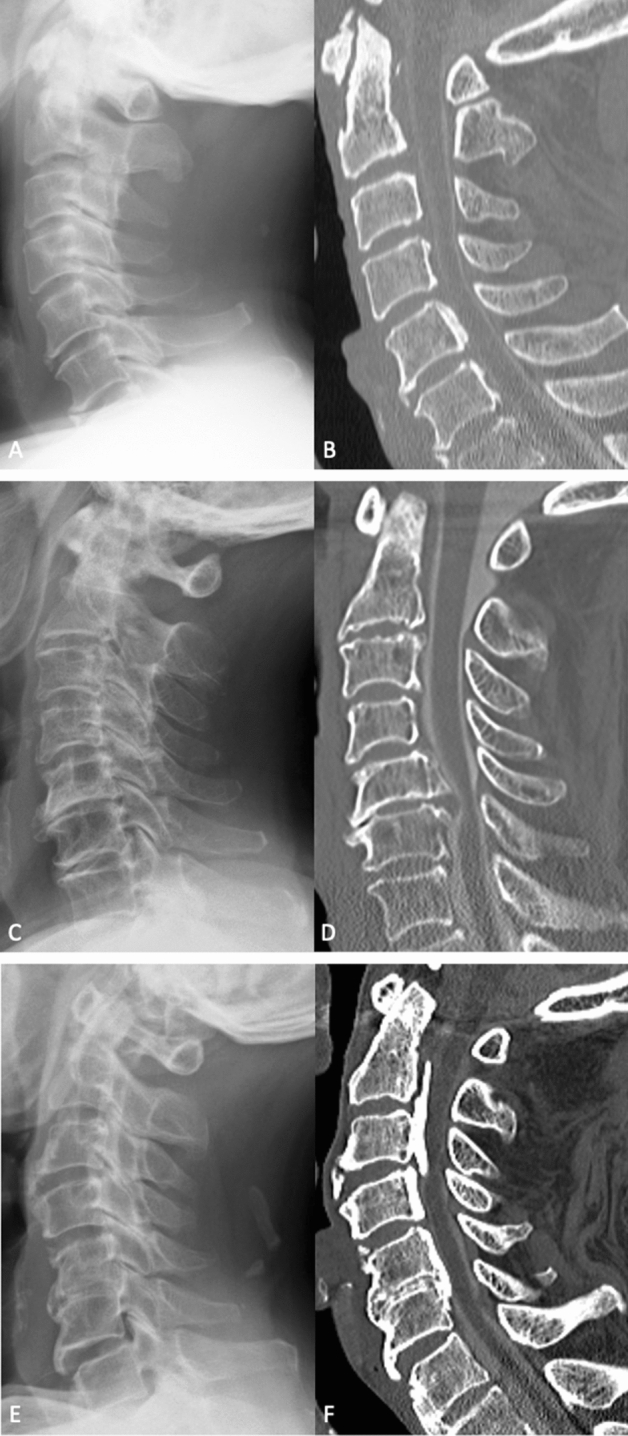
Representative radiographs used in the test dataset and their corresponding computed tomography (CT) images, for the reference of ossification of the posterior longitudinal ligament (OPLL). (**A**) Lateral radiograph of a patient with OPLL, which all spine surgeons misdiagnosed as cervical spondylosis, but the convolutional neural network (CNN) diagnosed correctly. (**B**) A CT scan of the same patient from panel A showing segmental OPLL in C5 and C6. (**C**) Lateral radiograph of a patient with OPLL, which all spine surgeons and the CNN misdiagnosed as cervical spondylosis. (**D**) A CT scan of the same patient from panel (**C**) showing segmental OPLL in C5 and C6. (**E**) Lateral radiograph of a patient with OPLL, which the CNN misdiagnosed as cervical spondylosis, but all spine surgeons diagnosed correctly. (**F**) A CT scan of the same patient from panel (**E**) showing a mixed type of OPLL in C2–C6.

The results and confusion matrix of 150 cases of the CNN are shown in Table 2. The accuracy, average recall, precision, and F1 score of the CNN and spine surgeons for classification of lateral cervical spine radiographs are presented in Table 3. The accuracy was higher for CNN compared to any expert spine surgeon, and was statistically equal to 4 of the 5 experts and significantly higher than that of 1 expert. The recall (sensitivity) of the CNN and spine surgeon for each OPLL type is presented in Table 4. The recall scores for segmental and localized types were lower compared to continuous and mixed types for both the CNN and spine surgeon groups. For reference, Fig. 1 shows representative lateral cervical spine radiographs and their corresponding CTs of OPLL, which the CNN and spine surgeons either diagnosed correctly or misdiagnosed.

**Table 2:** Confusion matrix of the CNN model.

		Prediction by the CNN			Total
		Cervical spondylosis	OPLL	Normal	
Ground truth	Cervical spondylosis	46	1	3	50
	OPLL	11	39	0	50
	Normal	4	2	44	50

*CNN* convolutional neural network, *OPLL* ossification of posterior longitudinal ligament.

**Table 3:** Performance metrics of the CNN and spine surgeons of diagnoses made using lateral cervical spine radiographs.

	Accuracy	*P* value (compared with CNN)	Average recall	Average precision	Average F1 score
CNN	0.86	–	0.86	0.87	0.87
Spine surgeon 1	0.83	0.50	0.83	0.82	0.83
Spine surgeon 2	0.83	0.43	0.83	0.83	0.83
Spine surgeon 3	0.81	0.22	0.81	0.81	0.81
Spine surgeon 4	0.81	0.20	0.81	0.84	0.83
Spine surgeon 5	0.76	0.018	0.76	0.76	0.76

*CNN* convolutional neural network.

**Table 4:** The recall (sensitivity) of the CNN and spine surgeon for each OPLL type.

OPLL type	Recall for the CNN (%)	Average recall for spine surgeons (%)
Continuous	83 (5/6)	81
Segmental	72 (13/18)	63
Mixed	87 (19/22)	94
Localized	50 (2/4)	63

## Discussion

This study showed that the ability of a CNN to distinguish between cervical spondylosis, OPLL, and control lateral cervical radiographs was equal or superior to that of expert spine surgeons. Segmental and localized OPLL types were difficult to diagnose by plain radiograph alone for both the CNN and the expert spine surgeons. Overall, this study demonstrated that CNN performance is promising and supports the possibility of an automated screening tool for OPLL.

Our CNN model successfully differentiated patients with OPLL from patients with cervical spondylosis and normal controls. This is the first study to evaluate the OPLL diagnostic ability of a CNN. Although there is consensus regarding the accuracy of detecting OPLL by CT, the reliability of plain radiographs to detect OPLL is inadequate compared to CT^6,8,10,11^. CT scans would improve the accuracy of diagnosis of OPLL, however, it is not feasible or reasonable to use CT in screening a large sample cohort^6^. The use of cervical radiographs as a screening tool is recommended in the nontraumatic setting for patients with local signs or symptoms such as motor or sensory deficits consistent with cervical root level distribution^12^. CT scans are also useful in determining OPLL type classification. Chang et al. investigated inter- and intra-observer agreement of Tsuyama’s cervical OPLL type classification^13^ on lateral plain radiographs and reconstructed CT images^8^. Inter- and intra-observer kappa values were only 0.51 and 0.67 for the lateral plain radiograph and 0.76 and 0.86 for 3D CT images, respectively. Kang et al. examined the diagnostic accuracy of cervical OPLL on lateral plain radiograph and magnetic resonance imaging (MRI) compared to CT scan. ﻿The diagnostic accuracy of lateral cervical radiograph and that of MRI were 52.2% and 58.7%, respectively^14^. In patients with a segmental or localized type of OPLL, the diagnostic accuracy of spine surgeons dropped to 27.3% and 20.0% respectively. They reported that in lateral cervical radiographs, localized and segmental types of OPLL were obscured by osteophytes, facets, and pedicles. Kudo et al. investigated the inter- and intra-observer reliability of the classification of OPLL types and diagnosis for OPLL using radiographs and CT images^10^. Inter- and intra-observer kappa values of the classification of OPLL type were 0.528 and 0.477 for the lateral radiograph and 0.633 and 0.605 for both radiographs and CT images, respectively^10^. Inter- and intra-observer kappa values of the diagnosis of OPLL were 0.743 and 0.613 for the lateral radiograph and 0.833 and 0.802 for both radiographs and CT images, respectively^10^. The diagnostic accuracy of the CNN was higher than reported in Kang et al., although a fair comparison is not feasible^14^. The present study also found it difficult to radiographically detect segmental and localized types of OPLL for both CNN and spine surgeons.

This study demonstrated that CNN is a promising screening tool for OPLL. Early diagnosis of OPLL, educating patients to avoid falls or trauma, and continued careful observation could lead to prevention of spinal cord injury and trauma-induced myelopathy^7^. Trauma in the neck can result in cervical spinal cord injury in patients with cervical OPLL^15,16^. It has been reported that 34% of traumatic cervical spinal cord injuries without bone injury were associated with cervical OPLL^5^. In 13% of OPLL patients who presented with myelopathy, trauma triggered the onset of myelopathy^7^. Nearly half of the patients who underwent surgery due to cervical myelopathy had fallen in the year before surgery^17^. Moreover, 37% of those who fell experienced worsening of motor deficits related to the fall, which were related to poor neurological outcomes^17^. Therefore, it is essential to diagnose OPLL at an early stage, educate patients to avoid trauma, and continue careful observation^7^.

There are several limitations to the present study. First, The validation dataset consists of an equal distribution of cervical spondylosis, OPLL, and normal controls and does not represent the prevalence of spondylosis and OPLL in the real world. However, the number of normal control images is limited because CT or MRI was required to confirm the absence of OPLL. Second, the present study did not include a class activation heatmap such as Grad-CAM as a visual explanation of the model^18^. Third, the CNN had a relatively small number of images. To conquer this problem, we applied transfer learning and data augmentation methods^19^. Although it was rare, the CNN missed obvious OPLL diagnoses as shown in Fig. 1E. Further investigations in larger cohorts are needed to improve the diagnostic accuracy of cervical spine OPLL. Finally, plain lateral cervical radiographs were acquired over the past 18 years and the image conditions were heterogeneous; however, since the accuracy of the CNN is thought to be improved by learning under various conditions, this may be a strength rather than a limitation^20^.

In sum, we showed that the ability of the CNN to differentiate between cervical spondylosis, OPLL, and normal cases using lateral cervical radiographs was equal or superior to that of spine surgeons. An artificial intelligence-based diagnostic model of lateral cervical spine radiographs could help non-experts diagnose cervical spine OPLL and also help determine whether further imaging is needed.

## Materials and methods

### Patients

The study was approved by the Institutional Review Board of the Chiba University Graduate School of Medicine and the requirement for consent was waived because of the retrospective analysis. (reference number 3329) All procedures involving human participants were in accordance with the 1964 Declaration of Helsinki and its later amendments. A retrospective review of the medical records of all patients who visited Chiba University Hospital between January 2003 and May 2020 was performed. Patients with cervical spondylosis, patients with OPLL, and patients with normal radiographic findings were enrolled. The cervical spondylosis group included patients who had been operated on in our hospital for cervical spondylotic myelopathy or cervical spondylotic radiculopathy. Cervical spondylosis refers to non-inflammatory disc degeneration, such as narrowing of disc height, vertebral body marginal hardening, osteophyte formation, Luschka joint deformity, and osteosclerosis of the facet joint surface^21^. Two orthopedic spine surgeons (MM, 7 years of experience and SM, 14 years of experience) confirmed the diagnosis of cervical spondylosis using both CT multiplanar reconstruction images and MRI. The OPLL group included patients who had been operated on in our hospital and patients who were followed up for observation after the diagnosis of OPLL was confirmed. OPLL was confirmed when two spine surgeons agreed on the diagnosis of OPLL based on CT scans. Most CT scans were acquired for surgical planning or for a definitive diagnosis of OPLL. Patients with normal radiographic findings were confirmed to have an absence of cervical spondylosis and OPLL using MRI or CT scans and were between 15 and 40 years of age^21–23^. In addition to radiographs, patients from this control group also received MRI or CT scans for examination of whiplash injury or neck pain, or for preoperative cervical spine screening for peripheral entrapment neuropathies such as cubital tunnel syndrome and carpal tunnel syndrome. Patients with cervical spondylotic myelopathy and patients with OPLL were evaluated for the Japanese Orthopaedic Association (JOA) scores at the time of lateral radiographs^24^. There were 250 patients with cervical spondylosis, 250 patients with OPLL, and 180 patients with normal radiographs. Exclusion criteria were cases with severe kyphotic deformity, atlantoaxial subluxation, previous cervical spine surgery, foreign body interference, obviously fused vertebrae, and cases with invisible C6 and C7 vertebrae.

### Radiological dataset

The dataset used in this study included lateral cervical spine radiographs in the neutral position of 311 cervical spondylosis cases, 269 OPLL cases, and 180 controls. We excluded 6 cases without CT, 5 cases with severe kyphosis, 2 cases with atlantoaxial subluxation, 45 postoperative cases, 2 cases with foreign body interference, 10 obviously fused vertebrae cases, and 1 case where both C6 and C7 were invisible. Moreover, 9 of the cases were excluded because both experts judged that OPLL and CS were difficult to distinguish even by CT due to disc calcification and osteophyte presence. Finally, a total of 80 cases, 61 cervical spondylosis and 19 OPLL, were excluded from the total patients list.

### Image preprocessing

**Figure 2:**
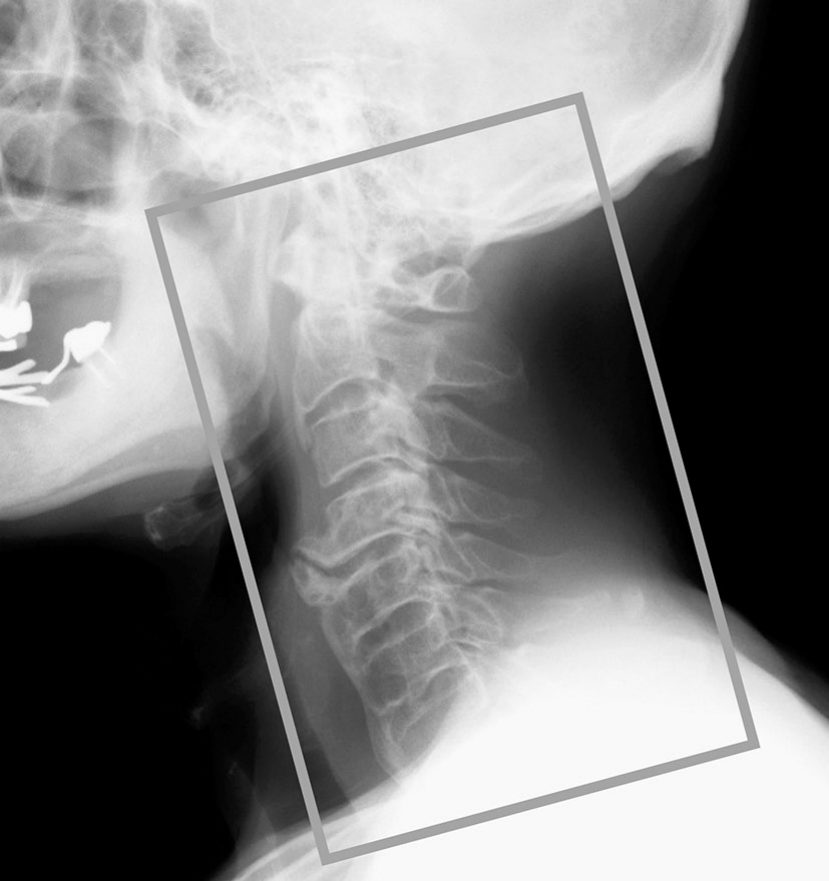
Image preprocessing for the convolutional neural network model training and validation. We cropped the smallest region with an aspect ratio of 2:3 (white box) containing vertebral bodies and spinous processes of C1–C7 in lateral cervical spine radiographs.

Plain lateral cervical spine radiographs were exported as a JPEG from digital imaging and communications in medicine (DICOM) files and the picture archiving and communication systems (PACS) in our hospital. An orthopedic surgeon (MM, 7 years of experience) used Paint 3D (Microsoft Corp, Redmond, WA, USA) to generate images for CNN training by cropping the smallest region with an aspect ratio of 2:3 containing C1–C7 of each lateral cervical spine radiograph (Fig. 2).

### Model construction and training of the CNN

The CNN architecture was built using Python Programming Language version 3.6.7 and Keras, version 2.1.6 with TensorFlow, version 1.12.0 (https://www.tensorflow.org) at the backend. In this study, we used the EfficientNetB4 architectural model, which had been previously trained using images with ImageNet^25^. The input images were scaled down to 380 × 380 pixels. EfficientNets is a group of image classification models developed based on AutoML and combined scaling. In EfficientNets, a simple, but highly effective composite scaling program is presented to enhance mobile-sized baseline networks to improve performance while maintaining efficiency. EfficientNet has fewer model parameters and is more accurate and efficient than existing convolutional networks. An EfficientNetB4 CNN with a single, fully connected 3-class classification layer was used. Then, we applied transfer learning to the model using the dataset of radiographs of cervical spondylosis, OPLL, and controls. The network was trained for 100 epochs with a learning rate of 0.1, and the learning rate decreased if no improvement was observed. Model training convergence was observed using cross-entropy loss. All images in the training dataset were augmented randomly using ImageDataGenerator (https://keras.io/preprocessing/image/) by a rotation angle range of 20°, width shift range of 0.2, height shift range of 0.2, and brightness range of 0.3–1.0. The CNN was trained and validated using a computer with a GeForce RTX 2060 graphics processing unit (NVIDIA, Santa Clara, CA), a Core™ i7-9750 central processing unit (Intel, Santa Clara, CA), and 16 GB of random-access memory.

### Performance evaluation

We evaluated the ability of the CNN model to distinguish cervical spondylosis, OPLL, and normal controls using a validation dataset that was not included in the training dataset. We trained the CNN model using 200 cervical spondylosis cases, 200 OPLL cases, and 130 normal cases. Then, we further validated the performance of CNN in an additional 150 cases using 50 cases in each group. For the 50 patients with OPLL in the test dataset, the type of the OPLL^13^ was also recorded. The same 150 test cases (50 cases in each group) were examined by 5 board-certified spine surgeons (KM, KM, HT, MM, and GI, 11, 11, 17, 17 and 21 years of experience, respectively) and their diagnostic accuracy was compared to that of the CNN. The spine surgeons were blinded to clinical information, such as patient age and sex.

### Statistical and data analysis

All statistical analyses were carried out using JMP Pro (version 14.2.0; SAS Institute Inc., Chicago, IL). We calculated the true positive (TP), true negative (TN), false positive (TP), and false negative (FN) rates based on the predictions of the CNN and spine surgeons. To assess performance, the mean values of accuracy, recall, precision, and F1 scores were calculated. Accuracy, recall, precision, and F1 scores were calculated by the following numerical formula; accuracy = (TP + TN)/(TP + FP + FP + FN + TN); recall = TP/(TP + FN); precision = TP/(TP + FP); F1 score = 2 × recall × precision/(recall + precision). Accuracy is a percentage of the correct predictions out of the total prediction made. Recall is a measure of the number of correct positive predictions from all positives in a dataset, also known as sensitivity. Precision is a measure for the correctness of a positive prediction and is also known as positive prediction value. F1 Score is the weighted average of precision and recall. Differences in the accuracy of diagnostic performance between CNN and spine surgeons were compared using the McNemar test.

## Data Availability

The datasets analyzed during the current study are not publicly available due to their containing information that could compromise the privacy of research participants.
